# The Water-Based Synthesis of Platinum Nanoparticles Using KrF Excimer Laser Ablation

**DOI:** 10.3390/nano12030348

**Published:** 2022-01-22

**Authors:** Oana Andreea Lazar, Călin Constantin Moise, Anastas Savov Nikolov, Laura-Bianca Enache, Geanina Valentina Mihai, Marius Enachescu

**Affiliations:** 1 Center for Surface Science and Nanotechnology, University Politehnica of Bucharest, 060042 Bucharest, Romania; oana.lazar@cssnt-upb.ro (O.A.L.); calin.moise@cssnt-upb.ro (C.C.M.); anastas.nikolov@cssnt-upb.ro (A.S.N.); laura.bianca@cssnt-upb.ro (L.-B.E.); geanina.mihai@cssnt-upb.ro (G.V.M.); 2 S.C. NanoPRO START MC S.R.L., Mitropolit Antim Ivireanu Street 40, 110310 Pitesti, Romania; 3 Institute of Electronics, Bulgarian Academy of Sciences, 72 Tzarigradsko Shousse Blvd., 1784 Sofia, Bulgaria; 4 Academy of Romanian Scientists, Splaiul Independentei 54, 050094 Bucharest, Romania

**Keywords:** Pt nanoparticles, laser ablation in water, KrF excimer laser, HR-STEM, UV/Vis

## Abstract

Our work presents, for the first time, a comprehensive study of the synthesis of fully metallic platinum nanoparticles (Pt-NPs) involving the ablation process in double distilled water using a KrF excimer laser. To obtain detailed information on Pt-NP morphology and optical properties, prepared colloids were characterized using High Resolution Scanning Transmission Electron Microscopy (HR-STEM) with advanced capabilities for Energy Dispersive X-ray Analysis (EDX), UV/Vis optical spectroscopy, and Direct Analysis in Real Time—Mass Spectrometry (DART-MS). The influence of the applied laser fluence and laser repetition rate (RR) values on the characteristics of the obtained Pt-NPs and the ablation process, respectively, were also analyzed. Spherical and spherical-like nanoparticles exhibiting aggregation were produced. The Pt-NP mean size values were between 2.2 ± 1.2 nm and 4.0 ± 1.0 nm, while their interplanar distance measurements showed a face-centered cubic (FFC) Pt lattice (111), as revealed by HR–STEM measurements, for all investigated samples. The smallest mean size of 2.2 nm of the Pt-NPs was obtained using a 2.3 J cm^−2^ laser fluence at a 10 Hz RR, and the narrowest size distribution of the NPs was obtained with a 2.3 J cm^−2^ laser fluence at a 40 Hz RR. A linear dependence of the Pt-NP diameters versus the laser repetition rate was found at a constant fluence of 2.3 J cm^−2^. The proposed eco-friendly synthesis route of Pt-NPs, because of its relative simplicity, has the potential for use in industrial production.

## 1. Introduction

Colloidal platinum nanoparticles (Pt-NPs) have attracted significant attention due to their physical and chemical properties, including stability, dispersion, size, shape, and morphology, which further determine their final applications, ranging from biotechnology to electronics. They may act as strong catalysts for the reduction of polluting gases generated by vehicles [1] or for the elimination of nitrous oxide generated in combustion processes [2], and they may enhance the catalytic activity for oxygen reduction reactions during the operation of proton exchange membrane fuel cells [1,3]. They have important applications in oxygen reduction reactions (ORRs) due to their remarkable electrocatalytic characteristics [4]. These reactions play a significant role in corrosion [5], water electrolysis [6], electrochemical energy conversion [7], diverse industrial processes [8], etc. Their strong catalytic abilities are used, for example, in direct methanol fuel cells (DMFCs) for the conversion of fuel at the anode and the reduction of oxygen at the cathode [9]. The extremely wide range of Pt-NP applications also includes coatings [10], textiles [11], nanofibers [12], plastics [13], magnetic nanopowders [14], multifunctional membranes [15], and cancer therapy [16,17]. Moreover, Pt-NPs are largely used in electronics manufacturing for the fabrication of internal electrodes of multilayer ceramic capacitors [1,18] or as conductor paste in conductive thick-film-integrated circuits [19]. Because of the wide range of applications in the nanotechnology of Pt material, further investigations can increase control over their properties.

Generally, noble metals such as platinum (Pt), gold (Au), silver (Ag), palladium (Pd), ruthenium (Ru), rhodium (Rh), and rhenium (Re) are attractive due to their remarkable properties, including the resistance against oxidation and other corrosive processes. A large range of methods to synthesize metallic nanoparticles is known, including microemulsion [20,21], radiolytic reduction [20,22], microwave irradiation [20,23,24], electrochemical synthesis [20,22,25,26], sonochemical reduction [20,24,27], chemical reduction [20,22,24,25,26,28], gamma irradiation [20,29], and pulsed laser ablation in a liquid medium [20,22,24]. Each of them has certain advantages, such as a narrow size distribution, but also disadvantages, such as the presence of residual, potentially toxic ions and the use of expensive equipment.

One new promising method is pulsed laser ablation in a liquid environment (PLAL). It represents a top-down approach, being a clean and versatile physical method. Moreover, it is easy to implement due to the relative simplicity of the experimental configuration, allowing for the preparation of pure and clean metallic colloidal nanoparticles with no use of harmful chemical reagents. Various clean metallic nanostructures with different characteristics (i.e., shape and size) can be fabricated by PLAL at room temperature with no use of a vacuum system [30,31,32]. Furthermore, surfactants can be added during the ablation process in order to prevent the agglomeration/aggregation of the nanoparticles. A few review articles describing the processes involved in PLAL have been published [32,33,34]. Several phenomena occur at the solid–liquid interface after the laser beam reaches the target surface: thermal evaporation, plasma plume and cavitation bubble formation, and condensation and nanoparticle growth, nd processes such as melting/boiling/explosions are possible for nanoparticles already created [35]. The laser beam parameters (pulse energy, fluence, and repetition rate), the target material and the used liquid medium are the main technological parameters in the PLAL technique. They can influence the physical and chemical properties of the ligand-free nanoparticles, and, by their adjustment, monometallic, bimetallic, alloy, or oxidized nanoparticles with desired sizes and shapes can be produced [36,37]. We will present here briefly some main points to explain the formation of nanoparticles in the case of ns-pulse duration. The interaction between the incident laser beam and the solid target at the solid–liquid interface gives rise to the creation of free electrons due to photoionization. These electrons absorb the photons of the laser beam and collide with target material ions. The heat-transformed energy of the laser beam causes processes such as melting and boiling, leading to vaporization where the laser beam meets the target. Thermal processes play a dominating role at a laser pulse duration greater than 10^−12^ s, as this time is adequate for the electron lattice interaction and generation of heat. The gas finally changes to plasma in the form of a plasma plume that consists of such ablated species as electrons, ions, radicals, and atoms originating from the target and ions from the liquid environment. It also absorbs a part of the laser beam energy and starts to expand in the direction perpendicular to the target surface. The adiabatic expansion of the plasma is supersonic in nature and is confined by the surrounding liquid. This results in the release of shockwaves in the liquid medium and stress waves in the solid target. Through the adiabatic expansion of a plasma plume from the ablated material, the plasma will cool down and will release the energy to the double distilled water (DDW). This causes the rapid vaporization of the liquid near the plasma and a bubble is formed, known as a cavitation bubble, which encompasses the ablated mass. This cavitation bubble expands in the liquid until it achieves a critical volume and subsequently starts shrinking. When it can shrink no further, the bubble collapses, and the NPs are released in the liquid in a jet-like structure. Not all of the free electrons that constitute the plasma recombine. As the plasma cools down, some of them attach to the NPs, affecting their agglomeration. These bubbles then play the role of reaction vessels, where the Pt-NPs are formed [38,39,40], and their water colloids are obtained.

Normally, during the PLAL fabrication of Pt-NPs, it should be remarked that, when the laser radiation interacts with water and the target immersed in it, three physical processes can take place: (i) interactions between the laser beam and the target, (ii) interactions between the laser beam and the liquid, and (iii) interactions between the laser beam and the Pt-NPs already created. They compete, and their relative impact depends on the technological parameters during the ablation process. By what has been said so far, this method used in the synthesis of metallic NPs has advantages, such as the fabrication of clean NPs at a small scale and the lack of a need to use toxic chemicals, and disadvantages, such as the high energy requirement, the low stability of the colloids, and the difficulty of controlling the diameter size and the shapes of the NPs (a wide size distribution) [41,42].

Many researchers have reported on the pulsed laser ablation of a Pt target in pure water by using an Nd:YAG laser in order to obtain metallic NPs with a small size and spherical shape. Most of them have fabricated different Pt nanostructures, where the mean size value of the NPs was around 10 nm. Mafune et al. prepared Pt-NPs in pure water and in a sodium dodecyl sulphate (SDS) solution using an Nd:YAG laser with a fundamental wavelength (λ = 1064 nm) and the second harmonic (λ = 532 nm). They observed that larger nanoparticles with an average diameter of 6.2 nm are obtained in pure water, while smaller nanoparticles with an average diameter of around 3 nm are obtained by using an SDS solution [43]. Moniri et al. ablated a Pt target in distilled water through an Nd:YAG laser with a fundamental wavelength at a 10 Hz repetition rate (RR) and a 2 J cm^−2^ laser fluence. The determined mean size value was found to be 18 nm [44]. In addition, the same group used PLAL to produce Pt-NPs in distilled water with an Nd:YAG fundamental wavelength (λ = 1064 nm) under an application of 10–40 V voltage between two gold electrodes. They found a smaller mean size value of 9 nm when an electric field was applied at 20 V/cm, while a larger mean size value of 20 nm was obtained without an electrical field [2]. Cueto et al. produced Pt-NPs in pure water by Nd:YAG laser with the fourth harmonic (λ = 266 nm) at RR of 10 Hz and laser fluence of 1.5 J cm^−2^ and they obtained a bimodal distribution of Pt-NPs with two mean size values of 1–4 nm and 6–8 nm [45]. Nichols et al. fabricated Pt-NPs in pure water, applying Nd:YAG with the third harmonic (λ = 355 nm) at laser fluences of 1–110 J cm^−2^, and their diameter size was between 1–30 nm [46]. Censabella et al. prepared Pt-NPs in deionized water, also by an Nd:YAG laser, and they obtained Pt-NPs with a mean diameter size of around 10 nm [47]. Bakhtiari et al. ablated Pt-NPs in distilled water using an Nd:YAG laser with the first harmonic (λ = 1064 nm) under the influence of an applied electrical field (0–50 V), and they obtained a smaller mean size value of 9 nm at 0 V/cm [48]. Condorelli et al. fabricated Pt-NPs in water and NPs already formed were ablated in polyynes by Nd:YAG with a fundamental wavelength. They obtained the small mean size value of 15 nm for the latter, while the mean size value for the Pt-NPs in water was between 15 and 20 nm [49]. Madlum et al. prepared Pt-NPs in DDW using Nd:YAG (λ = 1064 nm) with a changing number of laser pulses. They obtained Pt-NPs with a diameter size of 10 nm for 100 pulses and 20 nm for 150 pulses [50]. R. Fathima et al. obtained monometallic Pt-NPs and bimetallic Pt-NPs/Au by an Nd:YAG laser with the first harmonic. They found a small mean size value for monometallic Pt colloids of 18 nm, while this value was 15.5 nm for bimetallic material, determined using HRTEM microscopy [51]. Kohsakowski et al. ablated Pt-NPs in water by the PLAL process using a 3 ps laser system, and the Pt-NPs already formed were then added onto the C support by colloidal deposition. They obtained a small mean size value of 3.8 nm for 40 wt % Pt/C, while this value was 6.6 nm for 21 wt % Pt/C [52]. The above work was done using an Nd:YAG laser to synthesize Pt-NPs. There are only two works using a KrF excimer laser: by Yan et al., who obtained hollow Pt micro/nanoparticles (empty particles) in distilled water at different laser fluences (2.3, 3.6, and 6.8 J cm^−2^), where the diameters of the hollow spheres were found to be between 10 and 100 nm [53], and by Daria Riabinina et al., who synthesized complete Pt-NPs [54]. However, this work mainly deals with the synthesis of Pt-NP films obtained in a vacuum at different pressures of gases. Just one example of PLAL synthesis was used for a comparison of Pt-NPs deposited for film formation.

This study presents, for the first time, comprehensive experimental results regarding the synthesis of fully metallic Pt-NPs using a KrF excimer laser in DDW. The optimization of the process parameters, namely, the laser fluence and RR, was investigated to obtain the smallest nanoparticles’ mean diameter. The optical and morphological properties of the prepared Pt-NPs, including their shape, mean size, size distribution, and crystallinity, were investigated through different techniques, such as Ultraviolet/Visible Spectroscopy (UV/Vis), High Resolution-Scanning Transmission Electron Microscopy (HR-STEM), Energy Dispersive X-ray (EDX), and Direct Analysis in Real Time Mass Spectrometry (DART-MS). The Pt-NP mean size values were in the range of 2.2 ± 1.2 nm and 4.0 ± 1.0 nm. We found that the Pt-NP diameter increases linearly with the laser RR for a 2.3 J cm^−2^ laser fluence. The measurements of interplanar distances, via HR–STEM, showed a face-centered cubic (FFC) Pt lattice (111) for all investigated samples.

## 2. Materials and Methods

### 2.1. Pt-NP Synthesis

**Figure 1:**
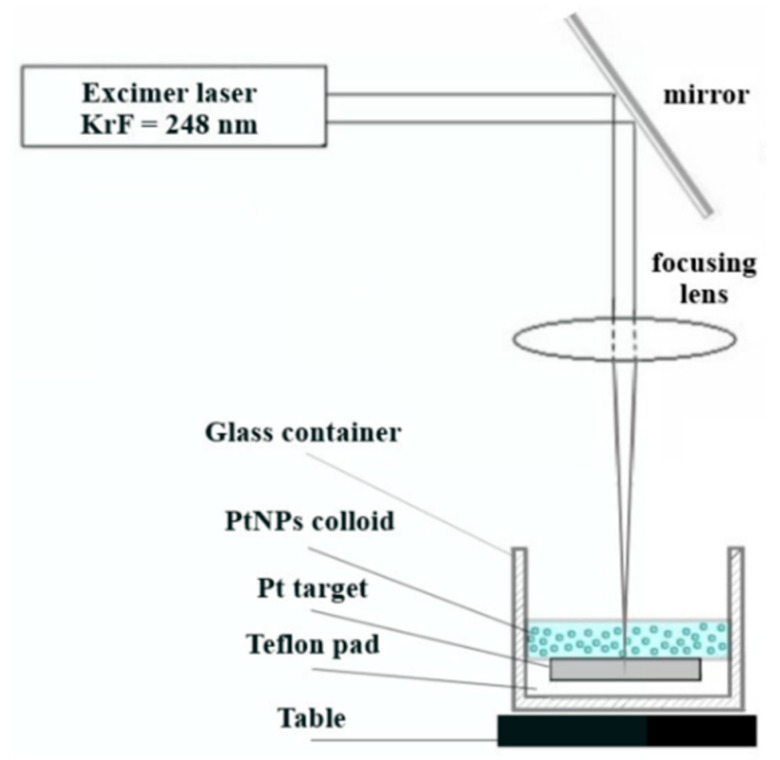
The experimental set-up of the pulsed laser ablation process in DDW.

Pt-NPs were fabricated using PLAL according to the experimental setup shown in Figure 1. The platinum target (99,95%) was a rectangular plate measuring 25 × 20 mm^2^ and a thickness of 2 mm. It was placed on a Teflon pad 5 mm thick, which in turn rested on the bottom of a cylindrical glass container. The Teflon pad touched the walls of the glass container, whose inner diameter was approximately 4.8 mm. The target was placed in a socket carved in the Teflon pad so that the upper surfaces of the target and the pad were on the same level. The groove of the socket was necessary to prevent uncontrolled sliding of the target in the direction of the walls of the glass container. Such relocation can cause the laser beam to fall unintentionally directly onto the Teflon pad and thus compromise the experiment by adding ablated material to the colloid. The glass container was placed on a horizontal table that could move vertically. Except for the Teflon pad, the experimental setup was similar to that employed in [55].

The volume of the liquid used in all experiments was 6.7 mL, which corresponds to a 4 mm height of the liquid above the upper surface of the target.

The source of laser irradiation was an excimer laser KrF COMPex Pro 205, with a wavelength of 248 nm and a pulse duration of 20 ns, produced by Coherent Inc. (Santa Clara, CA, USA). By means of a mirror, the laser beam was placed perpendicular to the surface of the colloid and to the target surface, respectively. The laser beam was focused using a biconvex quartz lens with a focal length of 300 mm placed in its path. The vertical movement of the table under the glass container allowed the laser spot on the surface of the target to lie in the focal plane of the lens or to move outside it. The distance between the edge of the lens and the upper surface of the target in our experiments was 304 mm. The dimensions of the rectangular laser spot were determined experimentally using photosensitive paper placed on the surface of the target. The laser spot in the focal point in air without liquid had a rectangular shape with an area of ~8.6 mm^2^. The ablation time was 15 min in almost all experiments. The ablation procedure was interrupted after 12 min only in the experiment with a 5.8 J cm^−2^ laser energy and a 50 Hz RR due to the scattering of the colloid outside the container.

Two parameters of the Pt-NP synthesis route varied in our study to optimize their characteristics: the laser fluence and the RR. Pt-NP samples were produced under constant laser fluences of 2.3, 4.0, and 5.8 J cm^−2^ for 5 different RRs of 10, 20, 30, 40 and 50 Hz, respectively.

### 2.2. Characterization of Pt-NPs

The optical and morphological properties of the Pt-NPs were studied using microscopic and spectroscopic methods.

The optical characteristics were determined by recording the transmission spectra of the prepared samples in the range 200–900 nm, using a Perkin Elmer Lambda 950 UV/Vis spectrophotometer equipped with a Peltier-controller PbS detector.

The morphological properties of the synthesized Pt-NPs, including their shape, mean size, size distribution, internal structure, and spatial organization (possible aggregation), were investigated through the HR-STEM technique using a Hitachi HD-2700 instrument operating at a 200 kV accelerating voltage equipped with an Energy Dispersive X-ray (EDX) Oxford Instruments X-max 100 TLE detector. Two kinds of electron images were acquired simultaneously, at the same magnification and in the same location on the sample, by using two different imaging techniques: the High Angle Annular Dark Field technique for ZC–phase contrast imaging and the Bright Field technique for transmission electron imaging. The compilation of the respective histogram and estimation of the average size of the nanoparticles from the TEM images was realized with the help of the program Image J. EDX was used to prove the platinum nature of the synthesized NPs.

The identification of the Pt atoms in the prepared samples was confirmed by AccuTOF LC—plus 4G MS from JEOL, Akishima, Japan, equipped with a DART ion source from IonSense Inc., Saugus, MA, USA.

## 3. Results and Discussion

The Pt-NP samples were labelled from S1 to S15, and the corresponding fabrication parameters used in PLAL are listed in Table 1: sample index, laser energy (mJ), RR (Hz), the total number of laser shots, fluence (J cm^−2^), the final volume of the colloid (mL), and lost volume during ablation. In all cases, the ablation time was 15 min, except for S15 as mentioned above. The initial volume of DDW was 6.7 mL.

In order to investigate the influence of the synthesis parameters on the Pt-NP characteristics, the study was organized into two parts: the effect of a changing RR at a constant fluence and the effect of a changing laser fluence at a constant RR.

### 3.1. The Effect of the Repetition Rate over Pt-NP Synthesis at a Constant Laser Fluence

**Figure 2:**
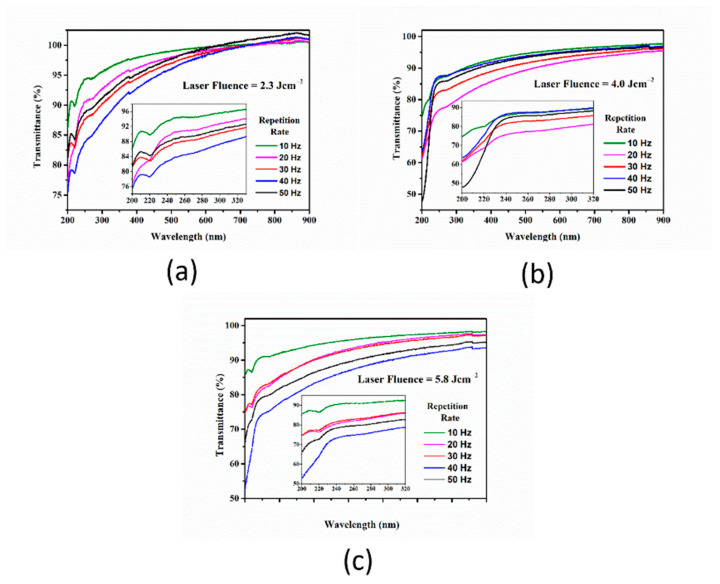
UV/Vis transmission spectra of prepared Pt-NPs at (**a**) 2.3 J cm^−2^, (**b**) 4.0 J cm^−2^, and (**c**) 5.8 J cm^−2^ for different RRs: olive at 10 Hz, magenta at 20 Hz, red at 30 Hz, blue at 40 Hz, and black at 50 Hz. The inset figure focuses on the 210–330 nm region.

The transmission UV/Vis spectra in the range 200–900 nm of the prepared samples with different RR values of 10, 20, 30, 40, and 50 Hz were acquired immediately after the PLAL process, and they are presented in Figure 2 for all three laser fluences used.

In all spectra having reproductible characteristics, two bands with a changed profile compared to the one in the neighboring regions can be distinguished. This is due to the lowered values of the transmission (enhanced extinction values). The first band, situated in the 200–230 nm interval, is clearly expressed with a certain local minimum at 220 nm. This band is more pronounced for the 2.3 J cm^−2^ and 5.8 J cm^−2,^ laser fluence families than in the 4.0 J cm^−2^ family. The position of this local minimum remains unchanged in all considered spectra.

**Figure 3:**
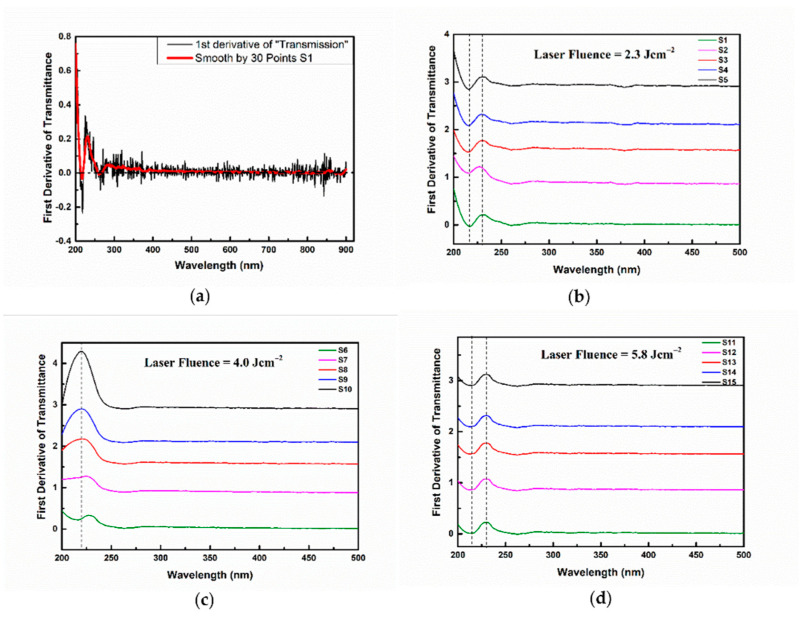
First derivative applied on UV/Vis transmission spectra from Figure 2: (**a**) representative graph of the first derivative applied on the S1 UV-Vis curve, (**b**) 2.3 J cm^−2^, (**c**) 4.0 J cm^−2^, and (**d**) 5.8 J cm^−2^.

Figure 3a represents the first-order derivative of the transmission spectrum of the S1 sample. As can be seen, the function presents some fluctuations (black curve). In order to be able to extract more precise information, a smoothing procedure was applied (red curve). The same procedure was applied to obtain the first-order derivative against the wavelength for all investigated samples. Figure 3b shows that the derivative of the UV/Vis transmission spectra from Figure 2a. Figure 3c corresponds to Figure 2b, and Figure 3d corresponds to Figure 2c.

The derivative function annulate for the 220 nm wavelength confirms the position of the transmittance minimum in the case of Figure 3b,d. The derivative locally presents a minimum and maximum at 215 nm and 230 nm, respectively. These shapes indicate an enhanced modification of the transmission at these wavelengths.

On the other hand, in Figure 3c, the derivative at 220 nm differs from zero, meaning that the transmission has no minimum at this wavelength (Figure 2b), as mentioned above, and the minimum at 215 nm also disappears for this fluence. The analysis of the first derivative proved to be useful.

The presence of this band is most probably due to the surface plasmonic resonance. This assumption is based on the results obtained in modeling the absorption spectrum of monodisperse platinum spherical nanoparticles with a diameter of 10 nm located in an aqueous colloid [45,56,57]. On the other hand, in the wavelength interval 230–330 nm, a very weakly expressed band with a lower transmission presenting unclear boundaries can be observed. The local minimum of this band is very weakly expressed in all considered spectra and positioned at about 270 nm. The first-order derivative annulate for this value confirms the position of the minimum of this peak. The presence of such a band is not observed in simulations calculated for monodisperse, well separated, spherical nanoparticles, as in [56]. This gives us reason to assume that the appearance of the band is rooted in the following possible factors: (a) presence in the colloid of individual, well separated nanoparticles with a diameter greater than 20 nm [58]; (b) presence in the colloid of elongated nanoparticles, i.e., with a shape deviating from the spherical one, in which longitudinal resonance can be observed [56,59]; (c) an effect of aggregation and the subsequent partial adhesion of nanoparticles and formation of different nanostructures. The explanation for this band requires further investigation. However, our transmission spectra are more pronounced and well-shaped than the spectra presented in other scientific works [43,46,53].

In all presented spectra, the transmission values in the whole interval at 20 Hz RR are lower than that at 10 Hz RR. In our opinion, this is due to the larger quantity of ablated material and, respectively, the larger mass concentration in the solution. In the case of the 2.3 J cm^−2^ family spectra, this tendency is preserved for 30 Hz and 40 Hz. Even though the 50 Hz spectrum does not satisfy this tendency, we decided to further analyze the sample S5 to obtain detailed information about the Pt-NP shapes at higher RRs. This is also useful for investigating the relationship between the Pt-NP diameters and the RR at a constant fluence. In all other cases of a higher RR and laser pulse fluences of 4.0 J cm^−2^ and 5.8 J cm^−2^, clear trends cannot be established. In our opinion, this is due to the occurrence of the optical breakdown leading to an uncontrollable process of ablation [60]. As can be seen in Table 1, the volume of the solution at the end of the ablation process drastically decreases (up to 56%, yellow highlight). It is appropriate to exclude such experiments from further study because of their unreliability. The excluded samples, S8, S9, S10, S13, S14, and S15, are highlighted in blue in Table 1.

**Figure 4:**
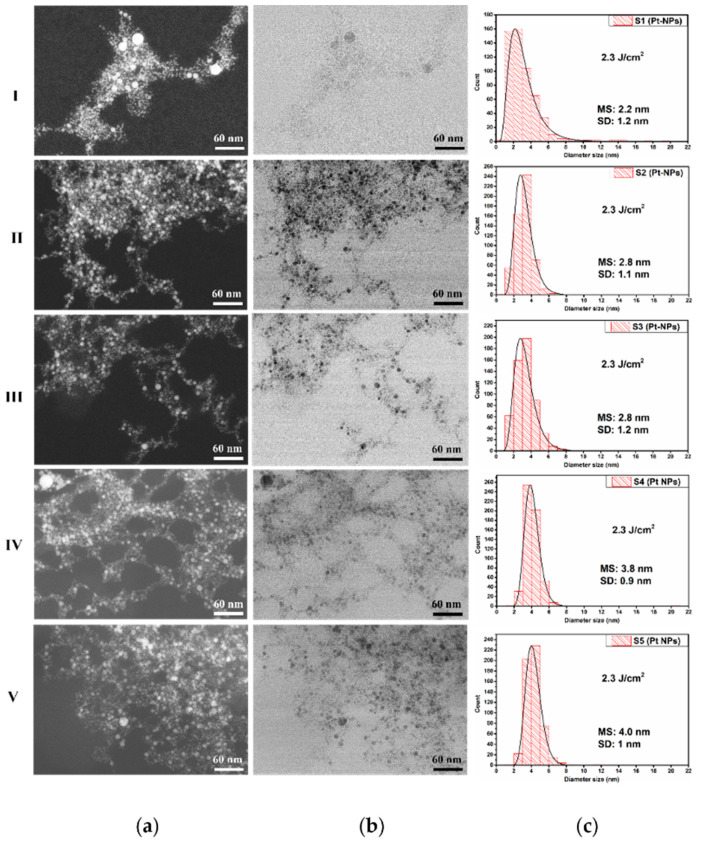
Pt-NP synthesized at a 2.3 J cm^−2^ fluence: (**a**) ZC images; (**b**) STEM images; (**c**) corresponding size distribution for laser RRs of (**I**) 10 Hz (S1), (**II**) 20 Hz (S2), (**III**) 30 Hz (S3), (**IV**) 40 Hz (S4), and (**V**) 50 Hz (S5), respectively.

Figure 4 shows the morphological characteristics of the samples prepared at a constant fluence of 2.3 J cm^−2^ for 10 Hz (I), 20 Hz (II), 30 Hz (III), 40 Hz (IV), and 50 Hz (V) using HR-STEM micrographs. Due to a special feature of our STEM, the ZC–atomic mass contrast (Figure 4a) and transmission images (Figure 4b) for every sample were simultaneously acquired at the same magnification (×500 K) and on the same location of the sample, being co-localized images.

The HR-STEM images revealed that the Pt-NPs have mainly a spherical and spherical-like shape, and many agglomerated, very small NPs are present. For good statistics, 550 individual Pt-NPs were measured with ImageJ software, and their histograms were fitted with lognormal distribution.

The smallest mean size value of 2.2 nm was determined in the case of Pt-NPs ablated at a 10 Hz RR, as can be seen in Figure 4(I-c). For a 20 Hz RR, the mean size value of the diameter increases at 2.8 nm (see Figure 4(II-c)). At a 30 Hz RR, the mean size value remains the same, as can be seen in Figure 4(III-c). However, for a 40 Hz and 50 Hz RR, the Pt-NP mean size values raise to 3.8 nm and 4.0 nm, respectively (Figure 4(IV-c,V-c)).

**Figure 5:**
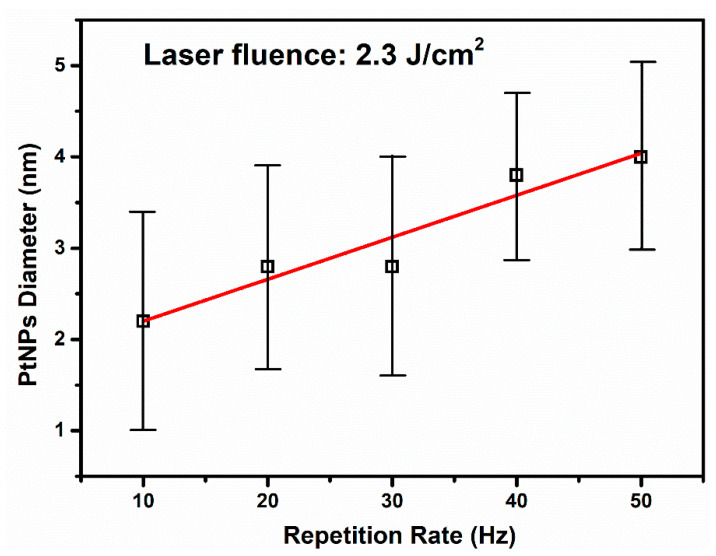
Pt-NP diameter size increases linearly with the repetition rate.

Two phenomena compete during the laser ablation of the target in a liquid, namely, the ablation of the target that generates nanoparticles and the irradiation of the colloid together with the Pt-NPs already produced. The irradiation of the NPs can induce fusion [54] (increasing their diameters); on the other hand, the smallest NPs can be melted [61]. Both events lead to an increase in Pt-NP diameter when the RR increases, i.e., increasing the energy transferred to the solution. We observed an increase of the mean size from 2.2 to 4.0 nm. In Figure 5, we show the Pt-NP mean size versus RR at a 2.3 J cm^−2^ laser fluence.

It is interesting that we observed a linear increase in the Pt-NP diameter’s mean size with the RR at a constant fluence. Such linearity dependence is confirmed by previous works on Au and Ag NPs [62,63]. However, to the best of our knowledge, this is first report of Pt-NP synthesis using a KrF excimer laser as the irradiation source.

The standard deviation values are not significantly affected by experimental conditions. They are similar in all investigated samples. The narrowest size distribution was obtained for the Pt-NPs fabricated at a 40 Hz RR.

**Figure 6:**
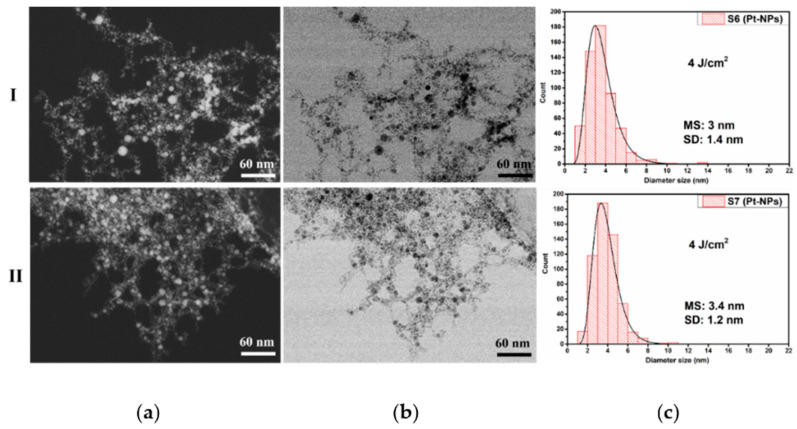
Pt-NP synthesized at a 4.0 J cm^−2^ fluence: (**a**) ZC images; (**b**) STEM images; (**c**) corresponding size distribution for the laser RRs of 10 Hz (S6) (**I**) and 20 Hz (S7) (**II**), respectively.

Figure 6 illustrates the co-localized ZC (a) and TEM (b) images of the Pt-NPs, including their morphological properties at a constant fluence of 4.0 J cm^−2^ for the 10 Hz (I) and 20 Hz (II) RR.

The HR-STEM micrographs show that all Pt-NPs have a spherical and spherical-like shape with a tendency of agglomeration. The sample S6 obtained with a 10 Hz RR shows a small mean size value of 3 nm and has a slightly wider size distribution of Pt-NPs (see Figure 6(I-c)). With the additional increase in the RR to 20 Hz, in S7, there is an increase in the mean size value to 3.4 nm, as shown Figure 6(II-c). As expected, these values are slightly larger than in the case of 2.3 J cm^−2^ at a 10 and 20 Hz RR. The standard deviation values are not significantly affected by the RR. The narrowest size distribution was obtained for the Pt-NPs fabricated at 20 Hz RR.

**Figure 7:**
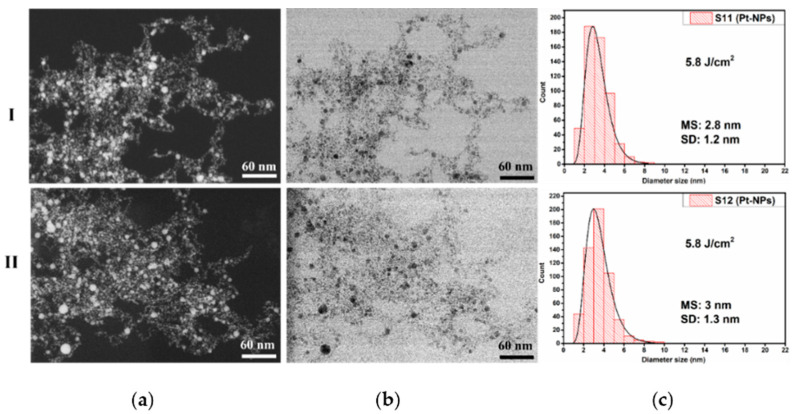
Pt-NP synthesized at a 5.8 J cm^−2^ fluence: (**a**) ZC images; (**b**) STEM images; (**c**) corresponding size distribution for laser RRs of (**I**) 10 Hz (S11) and (**II**) 20 Hz (S12).

Similar to the above figures, in Figure 7, the results for the S11 and S12 samples prepared at a constant fluence of 5.8 J cm^−2^ for 10 Hz (I) and 20 Hz (II), respectively, can be seen. The co-localized images are shown in Figure 7a (ZC) and Figure 7b (TEM).

The micrographs show, as in the previous case, that the Pt-NPs have mainly a spherical and spherical-like shape with a tendency of agglomeration. The sample S11 obtained with a 10 Hz RR exhibits a small mean size value of 2.8 nm and a narrow size distribution of Pt-NPs, which are agglomerated, as can be seen in Figure 7(I-c). With the additional enhancement of the RR to 20 Hz RR (S12), there is an increase in the average size value up to 3 nm with a somewhat wider size distribution (see Figure 7(II-c)). The relatively lower values of the mean size compared with 4.0 J cm^−2^ (namely, 3 nm and 3.4 nm, respectively) could be explained by the photodegradation process, whose probability increases as laser fluence rises. The width of the nanoparticle size distribution increases with RR enhancement. The narrowest size distribution was obtained for the Pt-NPs fabricated at a 10 Hz RR.

Summarizing, the smallest mean size value, i.e., 2.2 nm ± 1.2 nm (Figure 4(I-c)), was found for Pt-NPs synthesized at 2.3 J cm^−2^ with a 10 Hz RR. On the other hand, the narrowest size distribution was observed at the same laser fluence with a 40 Hz RR (Figure 4(IV-c)).

HR-STEM investigations indicated the presence of fully metallic Pt-NPs with mainly spherical and spherical-like shapes using different RR values. Finally, the fully metallic nature of the NPs and the smaller mean size, i.e., 2.2 nm ± 1.2 nm, obtained in our study differ from that reported in [2,43,44,45,46,47,48,49,50,51,52,53,54].

### 3.2. The Effect of Changing Laser Fluence for Pt-NP Synthesis at a Constant RR

**Figure 8:**
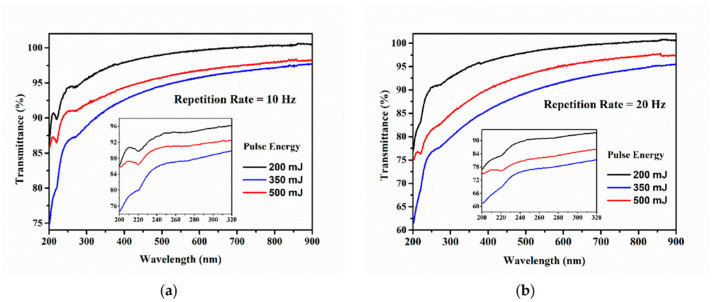
UV/Vis transmission spectra of Pt-NP samples produced with a 10 Hz (**a**) and 20 Hz (**b**) RR with different fluence values: black at 2.3 J cm^−2^ (S1 and S2, respectively), blue at 4.0 J cm^−2^ (S6 and S7, respectively), and red at 5.8 J cm^−2^ (S11 and S12, respectively). The inset figure focuses on the 210–330 nm region.

The transmission spectra of the colloids containing Pt-NPs under different laser fluence values while keeping the RR constant, as shown in Table 1 (S1, S6, and S11 at 10 Hz; S2, S7, and S12 at 20 Hz), are presented in Figure 8. All the spectra in Figure 8 are included in Figure 2 (not new data), and their features are commented on in Section 3.1, along with their first-order derivatives from Figure 3.

The highest transparency was found for the colloid fabricated with a 2.3 J cm^−2^ laser fluence (S1 and S2—black curve in Figure 8a,b). This is due to the relatively small quantity of ablated material and its low mass concentration in the colloid. At the laser fluence value of 4.0 J cm^−2^ (S6 and S7, blue curve in Figure 8a,b), the transmission decreases over the entire measured interval because of the increased amount of removed material from the target. The higher quantity of ablated material connected to the higher fluence is due to the enhanced transfer of laser energy to the target. A further increase of the laser fluence up to 5.8 J cm^−2^ (S11 and S12, red curve) shows a reversed tendency: the transmission value increases. In these conditions, the Pt colloids boiled, and an undetermined amount of material was scattered out of the beaker sustained by the decrease of the solution volume (i.e., 7.5% and 19%). It is also possible that some undefined quantity of Pt-NPs is ejected from the colloid together with the water. A similar slope was observed in the long wavelength region at 2.3 J cm^−2^ and 4.0 J cm^−2^ spectra, which is probably related to the formation of aggregates.

The size distribution of Pt-NPs synthesized at a 10 Hz RR for 2.3, 4.0, and 5.8 J cm^−2^ laser fluences corresponds to UV/Vis spectra shown in Figure 8a, along with the standard deviation values displayed in Figure 4, Figure 6 and Figure 7(I-c). The standard deviation of the obtained Pt-NPs is nearly the same for all three laser fluences, while the mean size values are 2.2, 2.8, and 2.8 nm. Changing the laser fluence from 4 to 5.8 J cm^−2^ does not affect the mean size of Pt-NPs. We suppose that the photodegradation of the NPs in the ablation process is responsible for this.

The mean size values of Pt-NPs obtained at a 20 Hz RR for all three laser fluence values are larger than those obtained with a 10 Hz RR, as can be seen in Figure 4, Figure 6 and Figure 7(II-c) (2.8, 3.4, and 3 nm). Most likely this is due to a greater quantity of ablated material and to the enhanced collision probability. The decrease of the mean size diameter in the case of 5.8 J cm^−2^ is also attributed to the photodegradation process during laser ablation.

### 3.3. HR-STEM Investigation for Individual Pt-NPs

**Figure 9:**
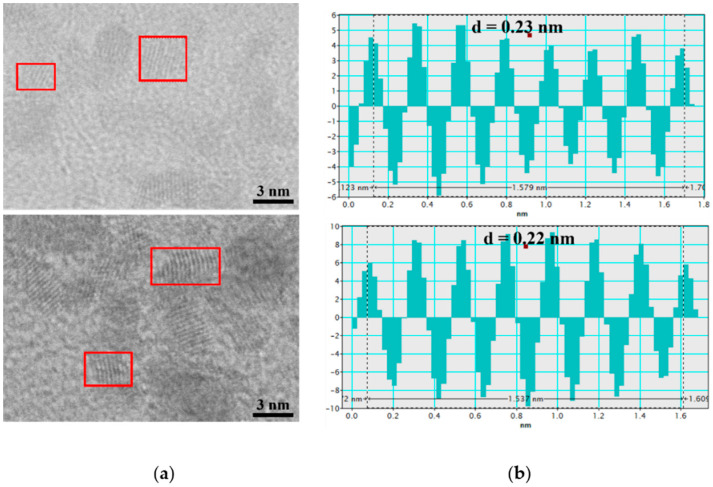
(**a**) HR-STEM image of synthesized Pt-NP colloids fabricated at a 10 Hz RR for S1 and S11; (**b**) corresponding profile for d-spacing measurement.

HR-STEM images of individual Pt-NPs, acquired at x8000K magnification (Figure 9a) demonstrates the spherical and spherical-like shape, crystallinity, and fully metal nature of NPs. By using such images, the corresponding profiles with d-spacing values for the investigated samples, S1 and S11, were measured (see Figure 9b). The interplanar distances of 0.22 nm and 0.23 nm (Figure 9b) for the crystalline planes of Pt (111) were then obtained from the red squares drawn in the transmitted electron images (Figure 9a). These values are almost identical to the ideal value of 0.23 nm for the platinum crystalline material with an FCC structure [64,65,66,67,68].

### 3.4. Confirmation of Platinum in the Colloids

**Figure 10:**
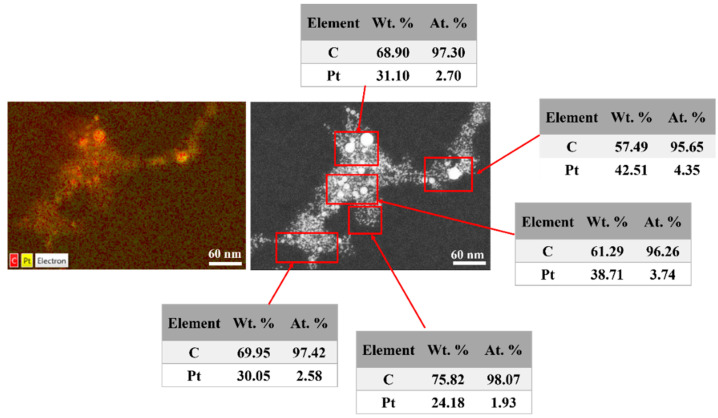
EDX analysis of Pt-NPs acquired at ×500K magnification: mapping analysis (**left** side) and elemental composition analysis (**right** side).

The EDX analyses carried out over the sample S1 at the same position with TEM analysis (Figure 4(I-a)) confirms that the nature of the NPs in the colloids is Pt. EDX is useful for identifying the elemental composition of the investigated sample. The EDX mapping of Pt-NPs is displayed in Figure 10 (left side), and the distribution of Pt-NPs on the Au grid can be seen in yellow. Since a thin layer of carbon exists on the surface of the gold grid for sustaining the investigated sample (Pt-NPs), the carbon distribution is also observed in red.

The elemental composition of the samples in atomic and weight percentages was determined from the EDX spectra corresponding to the areas indicated by red rectangles as shown in Figure 10 (right side). The average weight of the Pt-NPs was found to be around 33.31%, and the remaining 66.69% was attributed to the carbon material. The atomic average of the Pt-NPs was determined to be 3.06%, and the remaining 96.94% was determined to be carbon.

It is worth mentioning that the EDX performed for the smallest NP size (2.2 nm) confirms the presence of Pt in the colloids. DART-MS is a very sensitive tool for searching the presence of certain atomic mass in the sample, and we involved it as a complementary technique.

We used MS equipped with a DART source without any further sample preparation of the solution, and the presence of Pt-NPs was confirmed by a direct analysis of the colloid.

**Figure 11:**
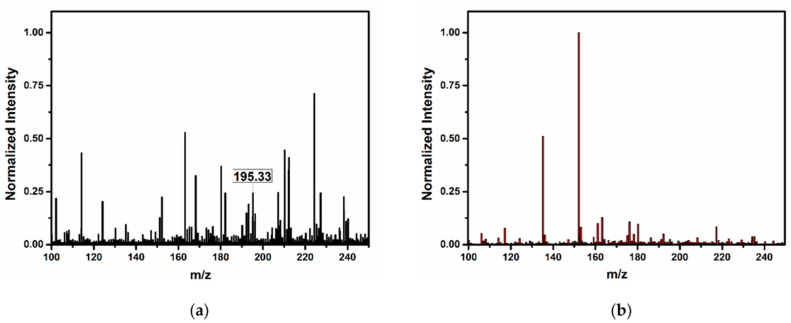
DART-MS spectra: (**a**) spectrum of the Pt-NP solution (**b**) spectrum of DDW.

The DART-MS spectrum of the investigated Pt-NP sample exhibits a visible line at 195.33 u (see Figure 11a), corresponding to the Pt-NP atomic mass (standard atomic mass for Pt is 195.09 u). On the other hand, the line of platinum is missing in the DDW spectra in Figure 10b, prior to the ablation process. This is direct proof of the presence of Pt in the colloid solution, which is also supported by the HR-STEM Pt lattice measurements and EDX analysis.

## 4. Conclusions

A comprehensive study of a fully metallic Pt-NP synthesis in DDW using KrF excimer laser ablation and its optimization was successfully achieved for the first time, to the best of our knowledge.

The impact of the laser parameters (such as laser fluence, laser RR, and the energy transferred to the liquid and target) over the fabrication process and the resulting Pt-NP characteristics were investigated.

UV/Vis spectroscopy was carried out immediately after each ablation process, and the obtained corresponding spectra were discussed.

The morphological properties were revealed using HR-STEM, and the electron images of fully spherical and spherical-like Pt nanoparticles were reported. The smallest mean size value, 2.2 nm ± 1.2 nm, was found for Pt-NP synthesized with the 2.3 J cm^−2^ laser fluence and a 10 Hz RR, and the narrowest size distribution of the NPs was obtained with the same fluence at a 40 Hz RR.

A linear increase of the Pt-NP diameter versus a laser RR was established, for the first time, at a 2.3 J cm^−2^ laser fluence supplied by a KrF excimer laser.

EDX analysis demonstrated the presence of Pt-NPs along with the HR-STEM interplanar distance measurements. The measured distances of 0.22 and 0.23 nm are specific to the crystalline planes (111) of Pt-NPs with an FCC structure. DART-MS was involved as a complement for Pt atomic mass identification in the colloidal solution.

The Pt-NP synthesis method presented here being relatively simple and also eco-friendly, could easily become an industry transfer.

## Figures and Tables

**Table 1:** The PLAL parameters used in synthesized Pt-NP samples.

Sample Index	E (mJ)	RR (Hz)	No. of Laser Shots	Fluence (J cm^−2^)	Final Volume (mL)	Lost Volume (%)
**S1**	200	10	9000	2.3	6.5	3
**S2**	200	20	18,000	2.3	6.5	3
**S3**	200	30	27,000	2.3	5.9	12
**S4**	200	40	36,000	2.3	5.9	12
**S5**	200	50	45,000	2.3	5.7	15
**S6**	350	10	9000	4.0	6.5	3
**S7**	350	20	18,000	4.0	6.2	7.5
**S8**	350	30	27,000	4.0	5.9	12
**S9**	350	40	36,000	4.0	5.1	24
**S10**	350	50	45,000	4.0	4.7	30
**S11**	500	10	9000	5.8	6.2	7.5
**S12**	500	20	18,000	5.8	5.4	19
**S13**	500	30	27,000	5.8	4	40
**S14**	500	40	36,000	5.8	3.5	48
**S15**	500	50	45,000	5.8	3	56

## Data Availability

The data presented in this study are available upon request from the corresponding author.
